# Do Variants in *GSTs* Modify the Association between Traffic Air Pollution and Asthma in Adolescence?

**DOI:** 10.3390/ijms17040485

**Published:** 2016-04-01

**Authors:** Gayan Bowatte, Caroline J. Lodge, Adrian J. Lowe, Bircan Erbas, Martine Dennekamp, Guy B. Marks, Jennifer Perret, Jennie Hui, Matthias Wjst, Lyle C. Gurrin, Katrina J. Allen, Michael J. Abramson, Melanie C. Matheson, Shyamali C. Dharmage

**Affiliations:** 1 Allergy and Lung Health Unit, Centre for Epidemiology and Biostatistics, School of Population & Global Health, the University of Melbourne, Melbourne, VIC 3053, Australia; gayan.bowatte@unimelb.edu.au (G.B.); clodge@unimelb.edu.au (C.J.L.); lowea@unimelb.edu.au (A.J.L.); jennifer.perret@unimelb.edu.au (J.P.); lgurrin@unimelb.edu.au (L.C.G.); mcmat@unimelb.edu.au (M.C.M.); 2 Murdoch Childrens Research Institute, Melbourne, VIC 3052, Australia; katrina.allen@mcri.edu.au; 3 School of Psychology & Public Health, Department of Public Health, Latrobe University, Melbourne, VIC 3086, Australia; b.erbas@latrobe.edu.au; 4 School of Public Health & Preventive Medicine, Monash University, The Alfred, Melbourne, VIC 3004, Australia; martine.dennekamp@monash.edu (M.D.); michael.abramson@monash.edu (M.J.A.); 5 Woolcock Institute of Medical Research, University of Sydney, Sydney, NSW 2037, Australia; guy.marks@sydney.edu.au; 6 South Western Sydney Clinical School, University of New South Wales, Sydney, NSW 2052, Australia; 7 Busselton Population Medical Research Institute, Perth WA 6009, Australia; jennie.hui@health.wa.gov.au; 8 Molecular Genetics of Lung Diseases, Comprehensive Pneumology Center, Helmholtz Zentrum, Muenchen 81377, Germany; wjst@helmholtz-muenchen.de; 9 Department of Allergy and Immunology, Royal Children’s Hospital, Parkville, VIC 3052, Australia

**Keywords:** asthma, *glutathione S-transferase*, hay fever, traffic related air pollution, wheeze

## Abstract

Polymorphisms in genes involved in the oxidative stress response may partially explain the documented heterogeneous associations between traffic-related air pollution (TRAP) exposure and asthma and allergies in children. We investigated whether the *GSTT1*, *GSTM1* and *GSTP1* gene polymorphisms modified the associations between TRAP exposure during the first year of life and asthma, wheeze and hay fever in adolescence. We used a birth cohort of 620 high risk infants from the Melbourne Atopy Cohort Study. TRAP exposure during the first year of life was defined as the cumulative length of major roads within 150 m of each participant’s residence during the first year of life. Wheeze, asthma and hay fever were measured at ages 12 (*n* = 370) and 18 (*n* = 434) years. The associations and interactions with *glutathione S-transferases* (*GST* s) were investigated using regression models. Overall, there was no relationship between TRAP exposure during the first year of life and current asthma, wheeze and hay fever at ages 12 or 18 years. However, in *GSTT1* null carriers, every 100 m increase in cumulative lengths of major road exposure during the first year of life was associated with a 2.31-fold increased risk of wheeze and a 2.15-fold increased risk of asthma at 12 years. TRAP is associated with some respiratory outcomes in carriers of genetic polymorphisms in oxidative stress metabolism genes.

## 1. Introduction

Asthma and other allergic diseases including allergic rhinitis, eczema and food allergy cause a substantial burden of disease in childhood. Although a rapid increase in asthma and allergies has been identified over the latter part of the 20th century, the reasons for this are still unknown [1]. Recent changes in environmental factors and their interactions with genetic profiles have been suggested as major factors responsible for the increase in asthma and allergic diseases [2,3]. Although there is a general consensus on the link between exposure to traffic-related air pollution (TRAP) and increased risk of asthma [2] the reported findings are not completely concordant [4]. This heterogeneity may be partly explained by genetic variation between populations conferring differences in susceptibility to TRAP exposure [5,6]. Further investigations are required to explore the role of genetic variants on the association between TRAP exposure and asthma and allergic diseases [4].

Many air pollutants behave as oxidants increasing oxidative stress among exposed individuals. It is plausible that this oxidative stress may trigger airway wall remodelling and increased risk of sensitisation [3,4]. Genetic polymorphisms in the *glutathione S-transferase* (*GST*) gene complex such as *GST mu1* (*GSTM1*), *GST pi1* (*GSTP1*) and *GST theta1* (*GSTT1*) are involved in antioxidant mechanisms. While these have been shown to modify the risk of allergic response to pollutants [7,8], the evidence for these interactions is inconsistent [9]. These inconsistencies may arise from differences between the studies, including, levels of air pollution exposure [10], age [11], sex [12] and ethnicity of participants [9,13].

Previous literature has shown that people carrying at least one *Val* allele in *GSTP1* have an enhanced risk of allergic sensitisation [14], asthma [11,14], wheeze [14,15] and impaired lung function [16], when exposed to TRAP. However, others have found that carriers of the *Ile* allele in *GSTP1* increased the risk of allergic diseases [10,17,18]. Population based studies reporting interaction of *GSTM1* polymorphisms for the association between TRAP exposure and respiratory diseases or allergies have shown no effect when considering the null genotype alone [14]. However, some studies have reported interaction effects for *GSTM1* evaluated jointly with other oxidative stress genes [12,19]. While the importance of *GSTT1* gene polymorphisms in influencing the development of asthma has been recognized [20,21], evidence concerning the modifying effect of *GSTT1* polymorphisms on the association between TRAP exposure and respiratory and allergic diseases has been published only twice, with both studies reporting null findings [22,23]. Past studies have investigated the interaction between *GST* genes and TRAP exposures on risk of asthma in birth cohort [14,15,22] and cross-sectional studies [10,11,18,23].

In this study, we aimed to investigate the modifying effect of *GST* gene polymorphisms on the relationship between TRAP exposure during the first year of life and the risks of asthma, wheeze and hay fever in adolescence using data from a high risk allergy birth cohort.

## 2. Results

**Figure 1:**
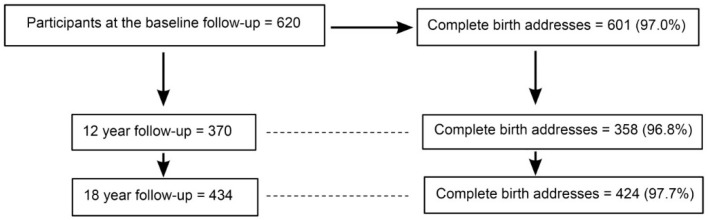
Melbourne Atopy Cohort Study follow-ups.

A flow diagram of the relevant study parameters from baseline to 12 and 18 years is presented in Figure 1. The baseline characteristics for the participants and non-participants at age 12 and 18 years are presented in Table 1. Those followed up to 18 years were more likely to have highly educated non-smoking parents. The prevalence of current asthma was 23.6% and 26.2% at 12 and 18 year follow-ups, respectively. Current hay fever prevalence increased from age 12 to 18 years (12 years, 37.4%; and 18 years, 50%). The prevalence of wheeze was 28.9% and 40.2% at 12 and 18 year follow-ups, respectively.

At the baseline, 601 participants had complete address data. Of the 601 participants who had complete address data, 23 moved to different addresses during the first year of life. However, of the 23 participants who moved during the first year, 14 did not participate at 12 and 18 year follow-ups. Of the 370 participants who participated at the 12 year follow-up, 296 participated in the 18 year follow-up. Our analysis included participants who resided in the same address during the first year of life and participated in the 12 and/or 18 year follow-ups (Figure 1). At baseline, 165 (27.5%) had any major road within the 150 m of their residence during the first year of life. There was no difference between exposure to major roads within 150 m, or genotype frequencies for participants and non-participants in any of the follow-ups (Table 1 and Table S1).

### 2.1. Main Environmental and Genetic Effects

Overall, TRAP exposure during the first year of life was not associated with any of the outcomes at 12 and 18 years (for exposure to cumulative length of major roads within 150 m or living ≤150 m from a freeway or highway of each participant’s residence during the first year of life) (Tables S2 and S3). None of the associations between *GSTs* and asthma, wheeze and hay fever were significant (Table 2). Some of the *GST* interactions for the association between cumulative length of major roads within 150 m and asthma and wheeze were significant (Table S4). However, none of the *GST* interactions were significant for the associations between living ≤150 m from a freeway or highway and any of the outcomes (Table S5).

### 2.2. Genotype Stratification and Interaction Effect of GSTT1

**Figure 2:**
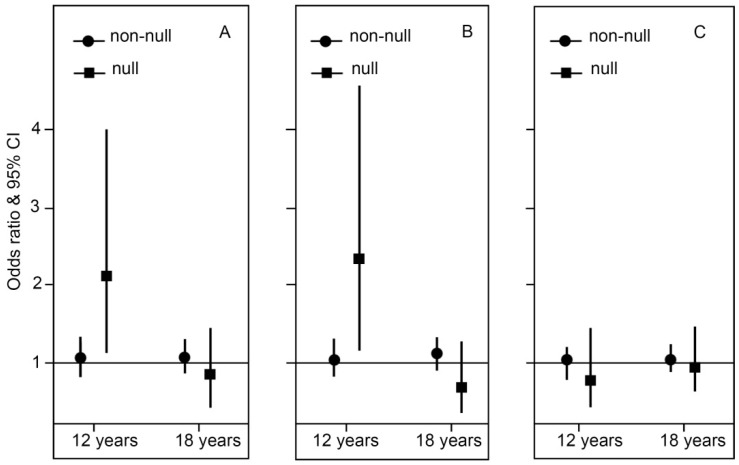
Association between traffic-related air pollution (TRAP) exposure during the first year of life and current symptoms of (**A**) asthma; (**B**) wheeze and (**C**) hay fever stratified by *GSTT1* polymorphism. Odds Ratios are for 100 m increase in cumulative lengths of major roads in 150 m buffer during the first year of life. Adjusted for parent asthma and smoking.

Carriers of the *GSTT1* null genotype had an increased risk of current asthma and wheeze at 12 years (OR 2.15 per 100 m increase in cumulative lengths of major roads within a 150 m buffer, 95% CI 1.15–4.00, *p* interaction = 0.04; and OR 2.31 95% CI 1.17–4.57, *p* interaction = 0.02, respectively), but not at 18 years. There was no interaction between *GSTT1* polymorphism and TRAP exposure during the first year of life for current hay fever at 12 and 18 years (Figure 2 and Table S4).

### 2.3. Genotype Stratification and Interaction Effect of GSTP1

**Figure 3:**
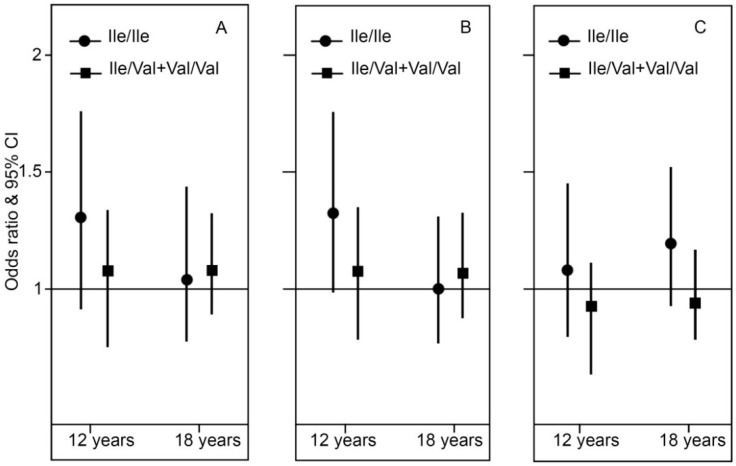
Association between TRAP exposure during the first year of life and current symptoms of (**A**) asthma; (**B**) wheeze and (**C**) hay fever stratified by *GSTP1* polymorphism. ORs are for 100 m increase in cumulative lengths of major roads in 150 m buffer during the first year of life. Adjusted for parent asthma and smoking.

Carriers of the *GSTP1* *Ile/Ile* genotype had a trend towards an increased risk of current asthma and wheeze at 12 years (Odds Ratio 1.28, per 100 m increase in cumulative lengths of major roads within a 150 m buffer, 95% CI 0.95–1.72, *p* = 0.10, *p* interaction = 0.26; and OR = 1.30 95% CI 0.97–1.74, *p* = 0.07, *p* interaction = 0.19, respectively), but not at 18 years. *GSTP1* polymorphisms did not modify the association between TRAP exposure during the first year of life and current hay fever at any time point (Figure 3 and Table S4).

### 2.4. Genotype Stratification and Interaction Effect of GSTM1

**Figure 4:**
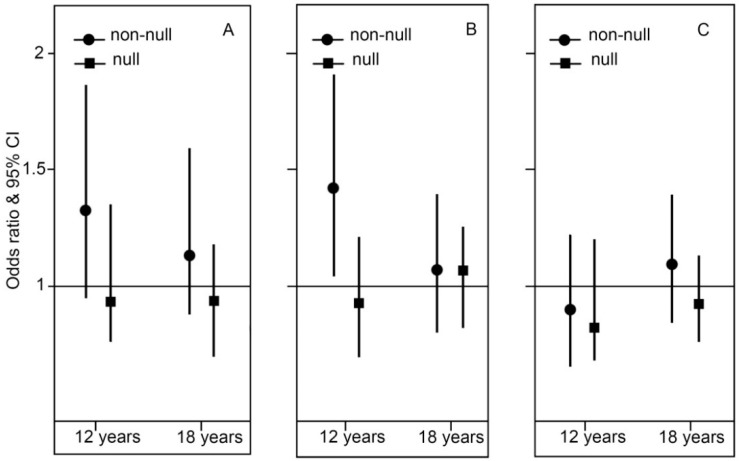
Association between TRAP exposure during the first year of life and current symptoms of (**A**) asthma; (**B**) wheeze and (**C**) hay fever stratified by *GSTM1* polymorphism. ORs are for 100 m increase in cumulative lengths of major roads in 150 m buffer during the first year of life. Adjusted for parent asthma and smoking.

Carriers of the *GSTM1* non-null genotype had a modest increased risk of current asthma and significant increased risk of wheeze at 12 years (OR 1.34, per 100 m increase in cumulative lengths of major roads within a 150 m buffer, 95% CI 0.98–1.83, *p* = 0.06, *p* interaction = 0.04; and OR 1.42 95% CI 1.05–1.92 *p* = 0.02, *p* interaction = 0.02, respectively), but not at 18 years. There was no interaction between *GSTM1* polymorphisms and TRAP exposure during the first year of life for current hay fever at any age (Figure 4 and Table S4).

## 3. Discussion

Our findings suggest that the association between TRAP exposure during the first year of life and early adolescent current asthma and wheeze may be modified by *GSTT1* and *GSTM1* gene polymorphisms. Carriers of *GSTT1* null and *GSTM1* non-null mutations when exposed to TRAP had an elevated risk of asthma and wheeze at 12 years. However, our study did not observe *GST* polymorphisms to modify the associations between TRAP exposure during the first year of life and hay fever. Australia is generally considered as a country with relatively low air pollution levels [24]. However, the findings of this study show that even at low levels, exposure to TRAP may be associated with a number of respiratory and allergic outcomes in carriers of genetic polymorphisms in these important oxidative stress metabolism genes.

This manuscript focused on the TRAP exposure during the first year of life and outcomes in adolescence because a recent review proposed that early childhood air pollution exposure and subsequent risk of asthma and allergies in later life can be explained by programming effects of air pollution exposures during early development on growing lungs and immune system. Air pollutants may impact structural and physiological functioning of the lung and changes in immune system. Further, these effects can be modified by genetic predisposition of the individuals [25].

Asthma is a chronic, heterogeneous disease and difficult to define until school age children. Further, asthma phenotypes during puberty, around 12 years is different from asthma phenotypes in young children and late adolescence [26]. This different type of asthma phenotype in puberty may be a reason for the observed significant interactions for asthma and wheeze at the age of 12 years but not at 18 years.

Our findings on the interaction between TRAP and *GSTT1* on asthma and wheeze have not been widely reported [9]. One study reported null findings for the interaction of *GSTT1* polymorphism for the cross-sectional association between TRAP exposure and adult asthma [22]. Another reported null findings for *GSTT1* interaction among children for particulate matter exposure from anthropogenic and non-anthropogenic sources and peak expiratory flow volume [23]. A recent meta-analysis including studies that investigated *GSTT1* polymorphisms and asthma found that regardless of TRAP exposure, carriers of *GSTT1* null mutation had an increased risk of childhood asthma [21]. To the best of our knowledge, ours is the first study to show TRAP exposure during the first year of life increasing risk of asthma and wheeze in late childhood among carriers of *GSTT1* null polymorphism. However, similar findings were not noted at the age of 18 years. Furthermore, these results were associated with unexpectedly large 95% CIs compared to other odds ratios from this analysis suggesting there may have been differential attrition at this time point that may explain this finding.

The role of *GSTs* in the oxidative stress pathway is well discussed in the literature. It is also accepted that air pollutants can produce reactive oxygen species (ROS) in exposed cells [9]. The null mutations of *GSTT1* and *GSTM1* represent deletion of genes responsible for enzyme production in the oxidative stress pathway. Therefore, carriers of *GSTT1* and *GSTM1* null mutations may have unbuffered ROS and higher levels of oxidative stress. These ROS may enhance airway inflammation resulting in asthma and wheeze [20]. Therefore, it is not surprising that those with null mutations are at increased risk of asthma when exposed to TRAP. However, in our study carriers of non-null mutation of *GSTM1* had an increased risk of wheeze at 12 years, when exposed to TRAP. Similar results were observed by David *et al.* 2003 [27] for the association between childhood ozone exposure and asthma. However, our findings for *GSTM1* were not concordant with most of the previous studies [9,28,29]. One explanation for these findings may be complex gene-gene interactions [27] from which we have taken only three genes into account.

Our findings that carriers of *GSTP1* *Ile/Ile* genotype had a modest risk of current asthma and wheeze at 12 years, although interaction was not significant, agree with the previous studies. Lee *et al.* 2004 [10] reported increased risk of childhood asthma in carriers of *Ile/Ile* alleles who lived in both low and high air pollution areas. Su *et al.* 2013 [18] also showed that carriers of *Ile/Ile* in *GSTP1* even living in areas with low concentrations of particulate matter less than 10 µm in diameter (PM_10_) had higher risk of childhood asthma.

One of the main strengths of this study was the longitudinal data in a well-defined birth cohort with multiple follow-ups to address long term associations. However, there were some limitations. We used total length of major roads within a 150 m buffer as a proxy for TRAP exposure but did not have information on individual air pollutants such as nitrogen dioxide (NO_2_) or particulate matter. A similar exposure method was developed and validated in Sydney, Australia where similar traffic conditions exist compared to current study area (Melbourne, Australia) and showed that road densities were strongly correlated with NO_2_ [30]. Additionally, we have not seen any associations when TRAP exposure was considered as living ≤150 m from a freeway or highway *vs.* living at a greater distance for the outcomes of asthma, wheeze or hay fever at 12 and 18 years. In this cohort very few participants (97/601) lived ≤150 m from a freeway or a highway. The small numbers in the exposed group may be a reason for not detecting any significant associations. On the other hand the total road length may be a better marker for TRAP exposure as it is a marker of total or cumulative road exposure that captures exposure to all roads nearby, which will contribute to the overall exposure levels [30]. Given the small numbers, especially in different strata, the results should be interpreted with caution. Findings of this study may also be more relevant to those with a family history of allergies than the general population. The Melbourne Atopy Cohort Study is predominantly Caucasian and Caucasian gene frequencies of null allele for *GSTT1* and *GSTM1* vary between 47% and 66% and 11% to 46%, respectively [31]. In the MACS cohort null gene frequencies for *GSTM1* and *GSTT1* were 58.5% and 17.5%, respectively. In healthy Caucasian populations *GSTP1* *Val* allele frequency is 14%–32% and in the MACS cohort it was 14% [32]. Although the MACS cohort is at high risk for allergic diseases the distribution of gene frequencies was within the published range.

In this cohort 56% and 36% of the participants lived in the birth address at the age of 12 and 18 years, respectively. Movement of the participants and change in air quality over the follow-up period can modify the associations between TRAP exposure during the first year of life and outcomes of asthma, wheeze and hay fever at 12 and 18 years. It has been shown that air quality of Victoria, Australia has gradually improved since 1990s’ [33]. However, we were unable to investigate these effects due to lack of availability of address data for other years. Additionally, these findings did not change after restricting the analysis to the participants who lived at the same address from birth up to 12 years (Table S6). In the current study we did not perform tests for correction of multiple comparisons. These corrections are made for studies investigating associations without pre-established hypotheses looking for significant associations. However, in this analysis genes were selected based on previously reported strong associations and we tested only predefined research questions. The idea of utilizing cohorts recruited as participants in a randomized controlled trial to test additional hypotheses about the relation between exposures observed and measured at baseline and outcomes ascertained during long-term follow-up is well established. It is based on the testable assumption that the randomized intervention will not influence the new associations of interest [34,35]. Past studies published from this cohort showed that the randomization status (infant formula allocation) was not associated with the outcome of interest and now this cohort continues as an observational study [36,37].

## 4. Materials and Methods

The Melbourne Atopy Cohort Study (MACS) started as a randomized controlled trial to investigate the association of infant feeding with the development of allergies [37]. Descriptions of enrolment and follow-ups are reported elsewhere [38]. Briefly, pregnant mothers from Melbourne, Australia were recruited during 1990–1994. A total of 620 infants with a family history of allergic diseases (asthma, eczema, allergic rhinitis, and/or severe food allergy) in a first degree relative were recruited during the antenatal period. The participants in this study are known as “probands”. The study was initially approved by the Mercy maternity hospital ethics committee (HREC no: R07/20 and R88/06 (19/1/1989)) and thereafter by Royal Children’s ethics committee for the 18 year follow-up (HREC: 28035 (14/9/2008)). The trial was retrospectively registered with the Australian and the New Zealand Clinical Trials Registry (ACTRN 12609000734268). All participants in the 18 year follow-up provided written informed consent.

### 4.1. Health Data Collection

A baseline questionnaire was administered before the birth of the babies which collected home environment and socioeconomic data. An allergy trained nurse conducted telephone interviews with the infant’s mother at 4 week intervals from birth to the age of 15 months and then at 18 and 24 months. Annual surveys were administered from 3 to 7 years of age. Clinical examinations were performed at 12 and 18 years. At all surveys before the 18 year follow-up, mothers answered questions regarding respiratory health and allergic conditions.

### 4.2. Definitions of Health Outcomes

Current asthma at 12 and 18 years: one or more episodes of asthma in the last 12 months and/or use of any asthma medications.

Current wheeze 12 and 18 years: any episodes of wheeze within the last 12 months.

Current hay fever at 12 and 18 years: one or more episodes of hay fever in the last 12 months and/or use of any treatment.

### 4.3. Genetic Data

Blood samples for genetic analysis were collected at the 18 year old follow-up (*n* = 429). *GSTM1* and *GSTT1* null genotypes were detected by a previously described multiplex PCR technique [39] and positive primers for beta-globin were included as a positive PCR control for all experiments. For *GSTM1*, individuals were categorised as either *GSTM1* null (homozygous for the GSTM1*0 allele) or *GSTM1* non-null (possessing at least one functional *GSTM1* allele). Individuals were categorised for *GSTT1* as either, *GSTT1* null (homozygous for the GSTT1*0 allele) or *GSTT1* non-null (homozygous or heterozygous for the GSTT1*1 allele). The *GSTP1* (rs1695 A→G: *Ile*105*Val*) polymorphism was genotyped using a customised Illumina GoldenGate Genotyping Assay (www.illumina.com). Individuals were categorised by genotype as *GSTP1-AA*, *GSTP1-AG* or *GSTP1-GG*.

### 4.4. Address Data and Exposure Assessment

Parent-reported residential addresses during the first year of life of the probands’ were geocoded. The sum of the length of major roads (freeways, highways, arterial and sub-arterial roads [40]) within a 150 m radius of the proband’s address in the first year was used as an index of TRAP exposure during the first year of life [30]. As a second TRAP exposure variable the distance from proband’s residential addresses during the first year of life to the nearest freeway or highway was also calculated. Then participants were categorized into two groups: (1) living ≤ 150 m and (2) living > 150 m. These lengths were calculated in ArcGIS 10.1 (ESRI 2012. ArcGIS Desktop: Release 10.1, Redlands, CA, USA: Environmental Systems Research Institute) using maps provided by the Victorian Department of Environment, Land, Water and Planning. The selection of 150 m for the buffer size was based on an observed decrease in pollutant levels with increasing distance from major roads [41]. Our major TRAP exposure was defined as the cumulative length of major roads within 150 m of each participant’s residence during the first year of life. Additionally, we defined TRAP exposure as living ≤150 m from a freeway or highway of each participant’s residence during the first year of life.

### 4.5. Statistical Analysis

The associations between exposure and categorical health outcomes were assessed using logistic regression models. In models the TRAP exposure during the first year of life was entered as a continuously-valued exposure variable for cumulative length of major roads within 150 m and as a binary variable for living ≤150 m from a freeway or highway.

Socio-economic status, parental smoking, parental history of asthma, cohort intervention (randomised to intervention or control group), keeping of furry pets in the household and gas cooking were identified as variables that could potentially confound the association between exposure and outcome. Potential confounders were retained in the final model if their inclusion changed the magnitude of the exposure-outcome association by more than 10% on the odds ratio scales for logistic regression. These analyses assume that the randomization factor of the cohort (infant formula allocation) does not affect the relationship between the exposures and outcomes studied within the analyses. This assumption was tested and there was no relationship between formula allocation and any of the outcomes considered in these analyses.

We also examined if the associations between TRAP exposure during the first year of life and our outcomes were modified by polymorphisms in the *GSTT1*, *GSTM1* and *GSTP1* genes. To do this we included an interaction term in the regression models. *GSTT1* and *GSTM1* genotypes were entered as a binary variable as either present or absent. *GSTP1* was also entered as a binary variable with the *Ile* allele defined as the risk allele and a recessive genetic model was assumed. All models included the same confounders identified as described above. Risks of diseases are reported as odds ratios and 95% confidence intervals (95% CI) per increase of 100 m in cumulative lengths of major roads in a 150 m buffer during the first year of life.

## 5. Conclusions

In conclusion, exposure to TRAP may affect lung health and allergic disease even in areas of low pollution for susceptible carriers of *GST* polymorphisms. Our findings provide further insight into the complex relationship between oxidative stress genes and TRAP exposure during the first year of life on asthma, wheeze and hay fever in adolescence. These findings are important from a public health perspective because carriers of certain polymorphisms of *GSTs* may be at higher risk of asthma and wheeze especially in children exposed to TRAP during the first year of life. Further studies are required to confirm the activity of enzymes related to *GST* genes.

## Figures and Tables

**Table 1:** Demographic data of participants and non-participants at baseline 12 and 18 year follow-ups.

Characteristics	During the First Year of Life	At 12 Years			At 18 Years		
	Baseline Participants (*n* = 620)	Participants (*n* = 370)	Non-Participants (*n* = 250)	*p*	Participants (*n* = 434)	Non-Participants (*n* = 186)	*p*
Female sex	303 (48.9%)	175 (47.3%)	128 (51.2%)	0.34 *	215 (49.5%)	88 (47.3%)	0.61 *
Mother’s education							
Primary	50 (8.0%)	24 (6.5%)	26 (10.4%)	<0.05 ^#^	30 (7.0%)	20 (10.5%)	<0.05 ^#^
Secondary	206 (33.2%)	113 (30.5%)	93 (37.2%)		129 (29.7%)	77 (41.4%)	
University	364 (58.7%)	233 (63.0%)	131 (52.4%)		275 (63.3%)	89 (47.8%)	
Father’s education							
Primary	68 (11.0%)	42 (11.4%)	31 (12.5%)	<0.05 ^#^	24 (5.5%)	30 (16.1%)	<0.05 ^#^
Secondary	172 (27.9%)	125 (33.8%)	65 (26.2%)		113 (26.0%)	61 (32.3%)	
University	377 (61.1%)	268 (72.4%)	152 (61.3%)		233 (53.7%)	92 (49.5%)	
Parental smoking	107 (17.5%)	75 (20.3%)	47 (19.1%)	0.71 *	63 (14.5%)	44 (24.0%)	<0.05 *
Parent asthma	379 (61.3%)	233 (63.0%)	146 (58.4%)	0.25 *	261 (60.0%)	118 (63.4%)	0.43 *
Living ≤150 m from a freeway or highway	97 (16.0%)	51 (13.8%)	46 (18.4%)	0.12 *	64 (14.7%)	33 (17.7%)	0.35 *
Major road length within 150 m buffer							
Median/m	261	261	262	–	262	254	–
Inter quartile range/m	102	105	92	–	110	122	–

Test for differences between the two populations: *z*-test * and *chi*-square test ^#^.

**Table 2:** Main genetic effects for asthma, wheeze and hay fever.

Gene Polymorphism	Outcome	12 Years			18 Years		
		Odds Ratio	95% CI	*p*	OR	95% CI	*p*
*GSTP1 rs1695*	Current asthma	1.16	0.66, 2.05	0.61	0.82	0.52, 1.29	0.39
	Current wheeze	0.82	0.49, 1.38	0.46	0.95	0.61, 1.49	0.83
	Current hay fever	1.14	0.70, 1.87	0.60	1.25	0.85, 1.86	0.26
*GSTT1*	Current asthma	0.79	0.36, 1.73	0.55	0.80	0.43, 1.50	0.49
	Current wheeze	1.31	0.67, 2.55	0.44	0.99	0.55, 1.78	0.97
	Current hay fever	0.74	0.38, 1.46	0.39	0.85	0.51, 1.43	0.55
*GSTM1*	Current asthma	1.00	0.57, 1.77	0.99	1.22	0.76, 1.94	0.41
	Current wheeze	1.28	0.76, 2.17	0.35	1.46	0.92, 2.32	0.11
	Current hay fever	0.92	0.56, 1.51	0.74	1.16	0.78, 1.72	0.47

In the regression model *GSTM1*/*GSTT1* null coded as “1” and *GSTM1*/*GSTT1* non-null coded as “0”. For GSTP1 *ile/ile* coded as “0” and *Ile/Val* + *Val/Val* coded as “1”. Adjusted for parent asthma and smoking.

## Supplementary Materials

Supplementary materials can be found at http://www.mdpi.com/1422-0067/17/4/485/s1.

## Abbreviations

*GSTs*	*Glutahione S transferaese* genes
*GSTT1*	*Glutahione S transferaese theta1*
*GSTM1*	*Glutahione S transferaese mu1*
*GSTP1*	*Glutahione S transferaese pi1*
MACS	Melbourne Atopy Cohort Study
NO_2_	Nitrogen dioxide
ROS	Reactive Oxygen Species
TRAP	Traffic Related Air Pollution
