# Supplementary material for: Do Variants in GSTs Modify the Association between Traffic Air Pollution and Asthma in Adolescence?
Source: Int J Mol Sci. 2016 Apr 1;17(4):485. doi: 10.3390/ijms17040485 (PMC4848941; doi:10.3390/ijms17040485)
Supplement: Supplementary file 1 [file ijms-17-00485-s001.pdf]

Supplementary Materials: Do Variants in *GSTs* Modify the Association between Traffic Air Pollution and Asthma in Adolescence?

Gayan Bowatte, Caroline J. Lodge, Adrian J. Lowe, Bircan Erbas, Martine Dennekamp, Guy B. Marks, Jennifer Perret, Jennie Hui, Matthias Wjst, Lyle C. Gurrin, Katrina J. Allen, Michael J. Abramson, Melanie C. Matheson and Shyamali C. Dharmage

Table S1. Genotype frequencies for *GSTT1*, *GSTP1* and *GSTM1* genes among 12 and 18 years participants and non-participants.

Genetic Polymorphism	At 12 Years			At 18 Years
	Participants	Non-Participants	*p*	Participants (%)
GSTT1 ($n = 428$)				
Non-null	249 (83.6%)	104 (80.0%)	0.40	353 (82.5%)
Null	49 (16.4%)	26 (20.0%)	0.40	75 (17.5%)
GSTP1 ($n = 429$)				
Ile/Ile	127 (42.6%)	57 (43.5%)	0.90	184 (42.9%)
Ile/Val	127 (42.6%)	58 (44.2%)	0.76	185 (43.1%)
Val/Val	44 (14.8%)	16 (12.2%)	0.45	60 (14.0%)
GSTM1 ($n = 424$)				
Non-null	131 (44.0%)	45 (35.7%)	0.11	176 (41.5%)
Null	167 (56.0%)	81 (64.3%)	0.11	248 (58.5%)

"*p*" Value for test for differences between the two populations—*z*-test. Note that blood samples were collected from participants at 18 years for gene testing; therefore non-participants not exist to compare.

Table S2. Associations of traffic-related air pollution (TRAP) exposure (cumulative lengths of major roads in 150 m buffer of each participant's residence during the first year of life) and asthma, wheeze and hay fever at 12 and 18 years.

Outcome	12 Years			18 Years		
	OR	95% CI	*p*	OR	95% CI	*p*
Asthma	1.02	0.86, 1.22	0.79	1.02	0.86, 1.21	0.80
Wheeze	1.03	0.87, 1.21	0.74	1.02	0.87, 1.21	0.77
Hay fever	0.89	0.75, 1.05	0.10	1.02	0.88, 1.18	0.80

ORs are for 100 m increase in cumulative lengths of major roads in 150.m buffer at home address during the first year of life. Adjusted for parent asthma and smoking.

Table S3. Associations between TRAP exposure (living ≤150 m from a freeway or highway of each participant's residence during the first year of life-categorical variable) and asthma, wheeze and hay fever at 12 and 18 years.

Outcome	12 Years			18 Years		
	OR	95% CI	*p*	OR	95% CI	*p*
asthma	0.78	0.36, 1.70	0.53	1.24	0.65, 2.36	0.52
wheeze	0.83	0.41, 1.69	0.61	1.09	0.58, 2.05	0.80
hay fever	0.59	0.30, 1.17	0.13	0.79	0.45, 1.37	0.39

Adjusted for parent asthma and smoking.

Table S4. Association between TRAP exposure (cumulative lengths of major roads in 150 m buffer of each participant's residence during the first year of life) during the first year of life and current symptoms of asthma, wheeze and hay fever stratified by *GSTT1*, *GSTP1* and *GSTM1* polymorphisms.

Genes	Outcome	*GSTT1* Non-Null				*GSTT1* Null				Interaction *p*	*n*
		OR	95%CI	*p*	*n*	OR	95%CI	*p*	*n*		
GSTT1	Asthma 12 years	1.03	0.82, 1.29	0.81	241	2.15	1.15, 4.00	0.02	40	0.04	281
GSTT1	Asthma 18 years	1.06	0.88, 1.28	0.52	323	0.78	0.43, 1.43	0.43	68	0.32	391
GSTT1	Wheeze 12 years	1.02	0.82, 1.26	0.88	240	2.31	1.17, 4.57	0.02	47	0.02	287
GSTT1	Wheeze 18 years	1.09	0.92, 1.30	0.33	332	0.66	0.34, 1.25	0.20	70	0.15	402
GSTT1	Hay fever 12 years	1.00	0.77, 1.17	0.63	234	0.77	0.42, 1.41	0.40	48	0.51	282
GSTT1	Hay fever 18 years	1.00	0.87, 1.19	0.83	328	0.95	0.63, 1.41	0.79	70	0.71	398
**Genes**	**Outcome**	***Ile/Ile***				***Ile/Val+Val/Val***				**Interaction *p***	***n***
		**OR**	**95%CI**	***p***	***n***	**OR**	**95%CI**	***p***	***n***		
GSTP1	Asthma 12 years	1.28	0.95, 1.72	0.11	120	1.01	0.76, 1.35	0.93	161	0.26	281
GSTP1	Asthma 18 years	1.00	0.76, 1.32	1.00	164	1.04	0.84, 1.29	0.74	228	0.84	392
GSTP1	Wheeze 12 years	1.30	0.97, 1.74	0.07	168	1.00	0.76, 1.31	0.99	119	0.19	287
GSTP1	Wheeze 18 years	0.98	0.74, 1.28	0.86	171	1.06	0.86, 1.30	0.61	232	0.64	403
GSTP1	Hay fever 12 years	1.05	0.79, 1.41	0.72	121	0.83	0.63, 1.08	0.17	161	0.24	282
GSTP1	Hay fever 18 years	1.16	0.91, 1.47	0.22	169	0.95	0.79, 1.14	0.55	230	0.19	399
**Genes**	**Outcome**	***GSTM1* Non-Null**				***GSTM1* Null**				**Interaction *p***	***n***
		**OR**	**95%CI**	***p***	***n***	**OR**	**95%CI**	***p***	***n***		
GSTM1	Asthma 12 years	1.34	0.98, 1.83	0.06	123	0.96	0.72, 1.30	0.81	158	0.04	281
GSTM1	Asthma 18 years	1.18	0.90, 1.55	0.22	164	0.92	0.73, 1.16	0.48	227	0.32	391
GSTM1	Wheeze 12 years	1.42	1.05, 1.92	0.02	128	0.93	0.71, 1.21	0.58	159	0.02	287
GSTM1	Wheeze 18 years	1.06	0.80, 1.39	0.69	168	1.02	0.82, 1.25	0.88	234	0.85	402
GSTM1	Hay fever 12 years	0.91	0.68, 1.22	0.53	124	0.92	0.70, 1.20	0.52	158	0.51	282
GSTM1	Hay fever 18 years	1.10	0.87, 1.38	0.44	165	0.94	0.77, 1.14	0.52	233	0.71	398

ORs are for 100 m increase in cumulative lengths of major roads in 150 m buffer at home address during the first year of life. Adjusted for parent asthma and smoking.

Table S5. Interactions of *GSTs* for the associations between TRAP exposure (living ≤150 m from a freeway or highway of each participant's residence during the first year of life) and current symptoms of asthma, wheeze and hay fever.

Genes	Outcome	Interaction *p*	*n*
GSTT1	Asthma 12 years	0.15	281
GSTT1	Asthma 18 years	0.67	391
GSTT1	Wheeze 12 years	0.08	287
GSTT1	Wheeze 18 years	0.45	402
GSTT1	Hay fever 12 years	0.42	282
GSTT1	Hay fever 18 years	0.75	398
GSTP1	Asthma 12 years	0.20	281
GSTP1	Asthma 18 years	0.52	392
GSTP1	Wheeze 12 years	0.24	287
GSTP1	Wheeze 18 years	0.60	403
GSTP1	Hay fever 12 years	0.33	282
GSTP1	Hay fever 18 years	0.16	399
GSTM1	Asthma 12 years	0.80	281
GSTM1	Asthma 18 years	0.90	391
GSTM1	Wheeze 12 years	0.94	287
GSTM1	Wheeze 18 years	0.97	402
GSTM1	Hay fever 12 years	0.59	282
GSTM1	Hay fever 18 years	0.88	398

Table S6. Associations of TRAP exposure (cumulative lengths of major roads in 150 m buffer of each participant's residence during the first year of life) and asthma, wheeze and hay fever at 12 and 18 years (restricted to participants who lived in the same address from birth to 12 years of age).

Outcome	12 Years			18 Years		
	OR	95% CI	*p*	OR	95% CI	*p*
Asthma	1.16	0.89, 1.50	0.27	1.09	0.80, 1.50	0.58
Wheeze	1.06	0.82, 1.37	0.66	1.17	0.87, 1.56	0.30
Hay fever	0.82	0.62, 1.09	0.17	1.13	0.86, 1.49	0.39

ORs are for 100 m increase in cumulative lengths of major roads in 150 m buffer at home address during the first year of life. Adjusted for parent asthma and smoking.
